# Effect of growth temperature on photosynthetic capacity and respiration in three ecotypes of *Eriophorum vaginatum*

**DOI:** 10.1002/ece3.3939

**Published:** 2018-03-06

**Authors:** Jessica L. Schedlbauer, Ned Fetcher, Katherine Hood, Michael L. Moody, Jianwu Tang

**Affiliations:** ^1^ Department of Biology West Chester University West Chester PA USA; ^2^ Institute for Environmental Science and Sustainability Wilkes University Wilkes‐Barre PA USA; ^3^ Biological Sciences The University of Texas at El Paso El Paso TX USA; ^4^ The Ecosystems Center Marine Biological Laboratory Woods Hole MA USA

**Keywords:** adaptational lag, *Eriophorum vaginatum*, moist tussock tundra, photosynthetic capacity, respiration, temperature acclimation

## Abstract

Ecotypic differentiation in the tussock‐forming sedge *Eriophorum vaginatum* has led to the development of populations that are locally adapted to climate in Alaska's moist tussock tundra. As a foundation species, *E. vaginatum* plays a central role in providing topographic and microclimatic variation essential to these ecosystems, but a changing climate could diminish the importance of this species. As Arctic temperatures have increased, there is evidence of adaptational lag in *E. vaginatum*, as locally adapted ecotypes now exhibit reduced population growth rates. Whether there is a physiological underpinning to adaptational lag is unknown. Accordingly, this possibility was investigated in reciprocal transplant gardens. Tussocks of *E. vaginatum* from sites separated by ~1° latitude (Coldfoot: 67°15′N, Toolik Lake: 68°37′, Sagwon: 69°25′) were transplanted into the Toolik Lake and Sagwon sites and exposed to either an ambient or an experimental warming treatment. Five tussocks pertreatment combination were measured at each garden to determine photosynthetic capacity (i.e., *V*_cmax_ and *J*_max_) and dark respiration rate (*R*_d_) at measurement temperatures of 15, 20, and 25°C. Photosynthetic enhancements or homeostasis were observed for all ecotypes at both gardens under increased growth temperature, indicating no negative effect of elevated temperature on photosynthetic capacity. Further, no evidence of thermal acclimation in *R*_d_ was observed for any ecotype, and there was little evidence of ecotypic variation in *R*_d_. As such, no physiological contribution to adaptational lag was observed given the increase in growth temperature (up to ~2°C) provided by this study. Despite neutral to positive effects of increased growth temperature on photosynthesis in *E. vaginatum*, it appears to confer no lasting advantage to the species.

## 1 INTRODUCTION

**Figure 1:**
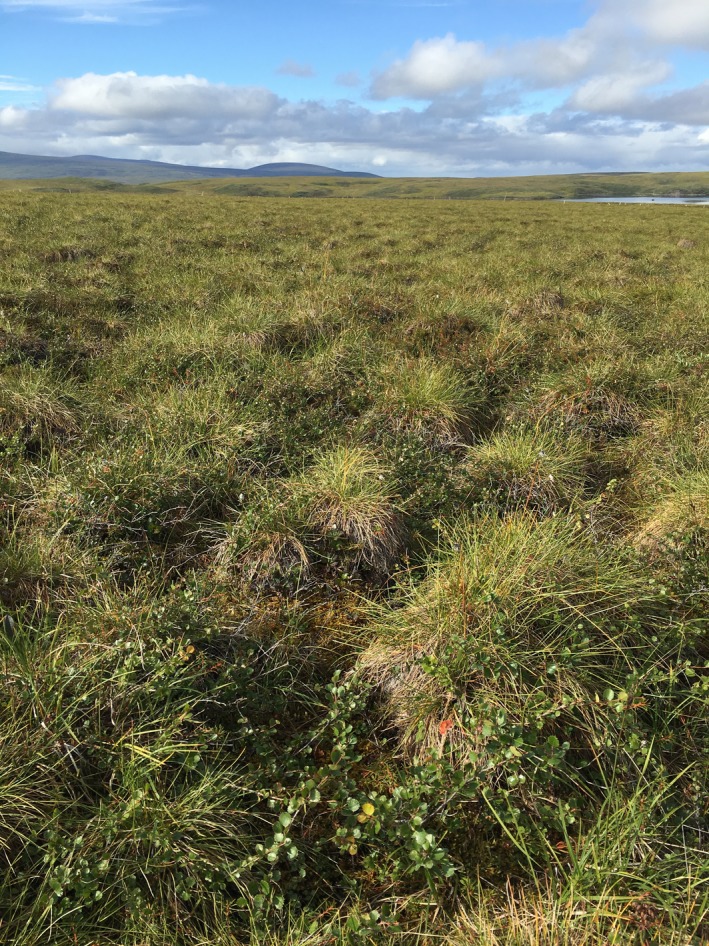
Moist tussock tundra at Toolik Lake, Alaska. The tussock‐forming sedge, *Eriophorum vaginatum*, is a foundation species in these ecosystems

Current and projected increases in global temperature are disproportionately affecting the Arctic (ACIA 2004; Diffenbaugh & Field, 2013), and ecosystem‐level changes in response to increased temperature include altered community composition, diversity, and productivity (Chapin, Shaver, Giblin, Nadelhoffer, & Laundre, 1995; Elmendorf et al., 2012; Oberbauer et al., 2007; Walker et al., 2006). Vegetation in northwestern North America's moist tundra is comprised of a mixture of graminoids, forbs, deciduous and evergreen shrubs, mosses, and lichens. The tussock‐forming sedge, *Eriophorum vaginatum*, is a foundation species for these ecosystems, providing much of the moist tundra's topographic structure, as well as microclimatic variation exploited by other plant species (Figure 1; Chapin & Shaver, 1985). Given *E. vaginatum's* central role in structuring these ecosystems, there is much interest in understanding the species’ response to a warming climate. Accordingly, these responses have been studied in reciprocal transplant gardens at six sites along a latitudinal gradient in Alaska, and it is evident that populations of this species form ecotypes that are locally adapted to climate (Bennington et al., 2012; Fetcher & Shaver, 1990; McGraw et al., 2015; Souther, Fetcher, Fowler, Shaver, & McGraw, 2014).

Differentiation of *E. vaginatum* into ecotypes has conferred “home‐site” advantage to populations, such that enhanced survival, tiller biomass, and light‐saturated photosynthesis have been found for ecotypes transplanted back into their home‐sites, relative to ecotypes from different latitudes (Bennington et al., 2012; Souther et al., 2014). While ecotypic differentiation is common in plants and often allows a species to occupy large and climatically variable niche space (Linhart & Grant, 1996), it may lead to adaptational lag (i.e., the rate of adaptation in a population may fall behind the rate of environmental change) if climate changes rapidly (Anderson, Panett, & Mitchell‐Olds, 2012; Etterson & Shaw, 2001). Because *E. vaginatum* is a long‐lived species (Mark, Fetcher, Shaver, & Chapin, 1985), ecotypes may have limited genetic variation and/or low gene flow between ecotypes, making the species susceptible to adaptational lag in a rapidly warming Arctic (Aitken, Yeaman, Holliday, Wang, & Curtis‐McLane, 2008). Evidence of adaptational lag is accumulating for *E. vaginatum*, as conditions for optimal population growth rates are now shifting north of a given ecotype's present location (Chandler et al., 2015; McGraw et al., 2015). This puts the species at risk for replacement by deciduous woody shrubs and could lead to substantial changes in ecosystem function (Heskel et al., 2013; Walker et al., 2006).

At present, little is known about possible physiological underpinnings to adaptational lag in *E. vaginatum*. Photosynthetic adjustments to growth temperature can manifest in a variety of ways (Way & Yamori, 2014), but the focus of this study is on whether variation in photosynthetic capacity contributes to adaptational lag in *E. vaginatum*. Measurement of the maximum carboxylation rate of Rubisco (*V*_cmax_) and maximum rate of electron transport (*J*_max_) allow for the assessment of photosynthetic capacity, as each are rate‐limiting photosynthetic processes (Sage & Kubien, 2007). Plants grown in cool climates acclimate by increasing the capacity of enzymes such as Rubisco, thereby enhancing photosynthetic capacity at low temperatures (Berry & Björkman, 1980; Yamori, Hokosaka, & Way, 2014; Yamori, Noguchi, & Terashima, 2005).

As growth temperature rises, it is unclear how photosynthetic acclimation may manifest in *E. vaginatum*; maintenance of low‐temperature enzymatic capacity would increase photosynthetic capacity at higher temperature, whereas a decline in enzyme concentration could lead to consistent or lower photosynthetic capacity across growth temperatures (Way & Yamori, 2014). Evidence of temperature homeostasis of photosynthesis between low and high growth temperatures in perennial herbs and woody evergreens adapted to cooler environments (Yamori et al., 2014) indicates that the latter option is likely, and “detractive adjustments” (after Way & Yamori, 2014) in photosynthesis with increased temperature sometimes occur (Berry & Björkman, 1980; Yamori et al., 2014). Additionally, declines in photosynthesis have been observed for a single *E. vaginatum* ecotype exposed to increased growth temperature (Heskel et al., 2013).

This study seeks to determine how the responses of *V*_cmax_ and *J*_max_ to increased measurement temperature vary for *E. vaginatum* ecotypes grown in transplant gardens under ambient and experimentally warmed conditions. Indications of adaptational lag in the species suggest that southern ecotypes will out‐perform locally adapted northern ecotypes as growth temperature increases. Variation in leaf morphology and leaf nitrogen content will also be investigated, as both can affect photosynthetic capacity. Additionally, the temperature response of dark respiration (*R*_d_) will be examined, as it has the potential to affect the overall carbon balance of *E. vaginatum*. Prior research indicates that while *E. vaginatum R*_d_ acclimates to higher growth temperature, *R*_d_ does not vary among ecotypes growing in common gardens (Heskel, Bitterman, Atkin, Turnbull, & Griffin, 2014; Kornfeld et al., 2013; Souther et al., 2014; van de Weg, Fetcher, & Shaver, 2013). This leads to an expectation that growing temperature‐induced changes in *R*_d_ will not differentially affect ecotypes, and any alterations in *E. vaginatum* carbon balance will be related to changes in photosynthetic processes. As a consequence, the following research questions were addressed: (1) Do *V*_cmax_ and *J*_max_ show evidence of detractive adjustments in photosynthesis as growth temperature increases? (2) Are detractive adjustments in photosynthesis more pronounced in northern ecotypes, relative to southern ecotypes? (3) Are responses of *R*_d_ to growth temperature uniform across ecotypes?

## 2 MATERIALS AND METHODS

### 2.1 Study species

*Eriophorum vaginatum* L. is a sedge that forms tussocks comprised of ~300–600 live tillers when mature (Fetcher & Shaver, 1982). Individual tillers produce 1–4 new leaves per year, exhibit deciduous root growth originating from tiller rhizomes, and typically live fewer than 8 years (Fetcher & Shaver, 1983). Tussocks grow vegetatively, with each tiller producing 1–3 daughter tillers per growing season (Fetcher & Shaver, 1983). Tussocks are thought to develop vegetatively from a single individual, and individual tussocks can persist for upwards of 100 years (Mark et al., 1985).

### 2.2 Experimental design

In 2014, tussocks of *E. vaginatum* from three ecotypes growing at the sites Coldfoot (CF, 67°15′32″N, 150°10′12″W), Toolik Lake (TL, 68°37′44″N, 149°35′0″W), and Sagwon (SAG, 69°25′26″N, 148°42′49″W) were transplanted into gardens located at TL and SAG. Coldfoot is located on the south slope of the Brooks Range, while both TL and SAG are north of the range and at a latitude above treeline. Ecotypes from these sites were selected because they represent a range of latitudes, each separated by ~1°.

**Figure 2:**
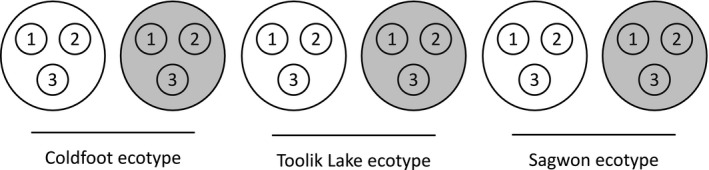
Schematic of transplant garden study design showing sets of three transplanted *Eriophorum vaginatum* tussocks (circles containing numbers) per ecotype and treatment (ambient—large white circles or open‐top chamber—large gray circles) combination. The pattern shown here was replicated 10 times at both the Toolik Lake and Sagwon gardens

Tussocks from each site were sliced from the ground beneath rhizomes to prevent significant damage during transplantation (Bennington et al., 2012; Parker, Tang, Clark, Moody, & Fetcher, 2017). Sixty tussocks per ecotype were transplanted into each garden. Tussocks from a given ecotype were transplanted in groups of three and assigned to either an ambient or an open‐top chamber (OTC) treatment, yielding 10 replicates per treatment (Figure 2). The OTC treatment was designed to passively warm tussocks, and chambers were constructed of fiberglass glazing (Sun‐Lite HP, Kalwall Corp., Manchester, NH) in an open‐ended cone shape. Chamber diameter was 1.23 m at the base and 0.84 m at the top. Chambers were 0.70 m in height and were secured with rope and tent stakes.

### 2.3 Field and laboratory measurements

To assess differences in light availability between treatments, photosynthetically active radiation (PAR, LI‐190SA quantum sensor, LI‐COR, Inc., Lincoln, NE) was measured every minute from 22 June to 23 August 2016 within one ambient and one OTC plot at the TL garden and recorded with a data logger (CR5000, Campbell Scientific, Inc., Logan, UT). The SAG garden was not instrumented for PAR measurements. Air temperature (*T*_air_) was measured hourly with shielded iButtons (DS1921G‐F5#, Maxim Integrated, San Jose, CA) mounted 20 cm above the ground surface at both gardens from 4 June to 27 August 2016. At TL, one sensor was located in an ambient plot and three were within OTCs, while at SAG, two sensors were in ambient plots and three were within OTCs.

To examine changes in photosynthetic rate (*A*) as a function of leaf intercellular CO_2_ concentration (*C*_i_; i.e., to develop *A*/*C*_i_ curves), gas exchange measurements were made on five tussocks per ecotype and treatment (i.e., ambient or OTC) combination at both the TL and SAG transplant gardens. A total of 30 tussocks were measured in each garden, and all measurements were made over a four‐week period from late June to late July 2016 between 0900 and 1700 Alaska Daylight Time (ADT, with one exception where a measurement ended at 1820 ADT). Alaska Daylight Time is 2 hr later than solar time. Measurements of gas exchange were made with two separate LI‐6400XT infrared gas analyzers (LI‐COR, Inc.), each using a 6400‐02B LED light source and 6400‐01 CO_2_ injector. Both analyzers were fitted with water jackets (6400‐88) on either side of the sensor head's Peltier coolers. Warmed or cooled water was circulated through the jackets as needed to help achieve target measurement temperatures of 15, 20, and 25°C within the LI‐6400's cuvette.

Prior to each measurement, six leaves per tussock were aligned parallel to one another and clamped into the cuvette. Given the small width of *E. vaginatum* leaf blades, leaf temperature (*T*_leaf_) within the cuvette was computed using the energy balance method during all gas exchange measurements. Leaves were cooled to 15°C, and measurement of an *A*/*C*_i_ curve was initiated with PAR set at 1,500 μmol m^−2^ s^−1^ for the measurement's duration. Twenty‐eight years of PAR data from TL show that mean hourly PAR exceeded 1,500 μmol m^−2^ s^−1^ for fewer than 0.6% of all hours in June and July (EDCT 2017), while light response curves showed that *E. vaginatum* leaves were light saturated, but showed no sign of photoinhibition at 1,500 μmol m^−2^ s^−1^ (data not shown). Photosynthetic rate was measured sequentially at the following ambient CO_2_ concentrations (*C*_a_): 400, 400, 300, 200, 100, 50, 400, 400, 600, 800, 1,000, 1,200 ppm, and measurements were initiated after stability was achieved in the cuvette (i.e., the rate of change in CO_2_ concentration, water vapor concentration, and flow rate over 15 s had a slope <1). The LI‐6400's block temperature was adjusted as needed to maintain *T*_leaf_ for the duration of each measurement, and cuvette relative humidity was maintained as close to 50% as possible. During *A*/*C*_i_ curve measurements, mean vapor pressure deficit (*D*) was 1.00 kPa at 15°C, 1.30 kPa at 20°C, and 1.89 kPa at 25°C. Following completion of each *A*/*C*_i_ curve, a measurement of *R*_d_ was made at the same temperature once stability had been reached, following ~5–10 min of darkness. Leaf temperature was next raised to 20°C and subsequently 25°C, and the above protocol was repeated at each measurement temperature on the same six leaf blades. When all gas exchange measurements were completed for a given tussock, the width of each leaf was measured in the field with an optical comparator, and gas exchange rates were recomputed based on the leaf area within the cuvette. Gas exchange data were not leak corrected.

In late July one to two tillers were clipped from an *E. vaginatum* tussock in each ecotype by treatment combination per garden, although at TL six of the 60 groups of tussocks were not sampled because of ongoing measurements that precluded removal of foliage. Collected leaves were stored at 4.5°C in plastic bags containing a damp paper towel for no longer than 48 hr. All leaves were scanned on a calibrated flatbed scanner and analyzed for leaf area with the program WinFOLIA (Regent Instruments, Inc., Montreal, QC, Canada). Leaves were then dried to constant mass at 70°C and weighed to determine specific leaf area (SLA). Dried leaves were ground into a fine powder with an oscillating ball mill (MM 200, Retsch, Haan, Germany) and packed into tin capsules for percent nitrogen (% N) analyses. Samples were analyzed at the Washington State University Stable Isotope Core Laboratory, and error analysis of duplicate samples representing 18% of all samples had a mean difference of 0.07% N. The % N and SLA data were used to compute nitrogen content per unit leaf area (N_area_, g N/m^2^).

Data from this study are archived at the Arctic LTER Data Archive, maintained by the Marine Biological Laboratory's Ecosystems Center (http://arc-lter.ecosystems.mbl.edu/content/ecotypic-variation-tundra-plants).

### 2.4 Data analyses

With the exception of the modeling described below, all analyses were carried out with R (v. 3.3.2, R Core Team 2016). Specific leaf area and N_area_ data were analyzed separately for each study site with ANOVA where the main effects were ecotype, treatment, and the ecotype × treatment interaction. To meet assumptions of normality, TL SLA data were 1/y transformed, while SAG SLA data were y^−2^ transformed. At SAG, two outliers >2 *SD* from the mean were removed prior to analysis of N_area_ data.

Individual *A*/*C*_i_ curves were fit using the R package plantecophys (v. 1.1.8, Duursma, 2015, 2016) based on the Farquhar, von Caemmerer, and Berry (1980) photosynthesis model where *V*_cmax_ and *J*_max_ were estimated at each measurement temperature, and the measurement of *R*_d_ at each temperature was used as an input variable. The Farquhar et al. (1980) model was fit with the parameterization of Medlyn et al. (2002), using the temperature functions of Bernacchi, Singsaas, Pimentel, Portis, and Long (2001) to determine the Michaelis‐Menten coefficients of Rubisco activity for CO_2_ (K_c_) and O_2_ (K_o_), as well as the CO_2_ compensation point in the absence of mitochondrial respiration (Γ*). The default fit method, employing nonlinear regression, was attempted for all *A*/*C*_i_ data. When the default method did not converge, a bilinear method was used to estimate *V*_cmax_ and *J*_max_. After curves were fit, the model fits and output data were examined to determine whether any data should be excluded from further analysis. Data were excluded if two of the four following criteria were met: (1) maximum *C*_i_ < 700 ppm, (2) photosynthesis did not plateau at high *C*_i_, (3) model RMSE > 6, or (4) the bilinear method was used to fit the curve. Criterion 1 ensured a reliable estimate of *J*_max_, while criterion 2 ensured that A had saturated. Criteria 3 and 4 were indicators of the quality of model fits. This process led to the removal of six of 90 *A*/*C*_i_ curves from the data set at the TL garden and three of 90 curves at the SAG garden. One additional tussock at SAG from the TL‐ambient treatment was removed from further analyses, as it was measured during an atypically cold period with *T*_air_ ~8°C, and residuals of *V*_cmax_, *J*_max_, and *R*_d_ measurements made for this tussock were at or beyond model standard error (see modeling details below).

The terms *V*_cmax_, *J*_max_, and *R*_d_ were modeled as a function of temperature with SigmaPlot (v. 11.0, Systat Software, Inc. 2008) as described below. Because *V*_cmax_ and *J*_max_ were measured over a relatively small temperature range, below the optimal temperature for each parameter, both *V*_cmax_ and *J*_max_ were modeled in relation to temperature with an Arrhenius equation (after Medlyn et al., 2002),

(1) $$f\left(T_{k}\right)=k_{25}\text{exp}\left[\left(E_{a}\left(T_{k}-298\right)\right)/\left(298\;R\;T_{k}\right)\right]$$

where *f*(*T*_k_) represents either *V*_cmax_ or *J*_max_ at a given measurement temperature, k_25_ is the value of either *V*_cmax_ or *J*_max_ at 25°C (hereafter referred to as *V*_cmax_(25) or *J*_max_(25)), *E*_a_ is the activation energy (exponential rate of increase) for either *V*_cmax_ or *J*_max_, R is the gas constant (8.314 J mol^−1^ K^−1^), and *T*_k_ is measurement temperature in Kelvin. Modeling was carried out separately for each ecotype by treatment combination at each garden.

The ratio of *J*_max_ to *V*_cmax_ at 25°C was computed for each tussock and analyzed with a three‐way ANOVA to determine whether the ratio varied across gardens, ecotypes, and/or treatments. The ANOVA yielded no significant differences in any of the main effects or in any of the interaction terms (*p* > .05); therefore, data were pooled across gardens, ecotypes, and treatments to examine the relationship between *J*_max_ and *V*_cmax_ with linear regression.

To assess the effect of the largest difference in growing season temperature imposed by this study on net photosynthesis (*A*_net_), data from the TL garden were examined. As described below, the OTC treatment effect at TL was stronger than at SAG, and both common gardens had nearly identical ambient growing environments in 2016. Therefore, separate ANOVAs were performed for each ecotype growing at the TL garden to determine the effect of treatment, measurement temperature, and their interaction on *A*_net_. Data for these analyses were extracted from *A*/*C*_i_ curves using the final measurement made at a CO_2_ concentration of 400 ppm. Data for the TL ecotype were y^−1/2^ transformed to meet assumptions of normality.

Variation in *R*_d_ as a function of temperature was modeled for each ecotype by treatment combination within a garden with the equation,

(2) $$R_{d}=a\text{exp}\left(b\;T_{\mathrm{leaf}}\right)$$

where *a* and *b* are modeled coefficients (Lloyd & Taylor, 1994). The temperature responsiveness (i.e., *Q*_10_) of each ecotype by treatment combination was determined with the equation,

(3) $$Q_{10}=\text{exp}\left(10\;b\right)$$

where *b* is the coefficient determined in Equation (2). Dark respiration was modeled using an exponential function rather than a variable *Q*_10_ function because measurements were made over a small temperature range (i.e., 10°C) and were made below the optimal temperature for *R*_d_ (Atkin, Bruhn, & Tjoelker, 2005).

Finally, because photosynthetic temperature responses can be confounded by stomatal closure as temperature, thus *D*, rises, stomatal conductance (*g*_s_) was compared to *D* using separate linear regressions for each ecotype. These data were extracted from *A*/*C*_i_ curves as described above.

## 3 RESULTS

### 3.1 Meteorological site comparison

Fifteen years of *T*_air_ data for the months of June, July, and August show that, of the three sites from which *E. vaginatum* ecotypes were collected, CF had consistently warmer mean, minimum, and maximum *T*_air_ than TL or SAG (Table 1). Mean monthly *T*_air_ was 3.2–6.1°C warmer at CF than at TL or SAG, depending on month and site (Table 1). Over the last 15 years, growing season *T*_air_ was similar for TL and SAG, with mean *T*_air_ slightly warmer (0.6°C) at TL in June, nearly identical in July, and slightly warmer (0.4°C) at SAG in August (Table 1). During the 2016 growing season, patterns in *T*_air_ were similar to those over the last 15 years, with CF as the warmest site and a high degree of similarity between TL and SAG (Table 1). Mean thawing degree days (TDD, the sum of daily mean *T*_air_ >0°C between May and September, after Shaver, Fetcher, & Chapin, 1986) computed for the past 15 years indicate that CF had the highest TDD (1606 ± 37 (*SE*)), followed by TL and SAG, which were statistically indistinguishable (TL: 944 ± 27, SAG: 971 ± 37) (EDCT 2017, NRCS 2017).

**Table 1:** Mean, minimum, and maximum temperature (*T*_mean_, *T*_min_, *T*_max_) averaged monthly ±*SE* for June, July, and August at the study sites from which tussocks were transplanted. Data for the 15 year period from 2002 to 2016 are presented along with those from just the 2016 growing season. Data from Coldfoot and Sagwon are from SNOTEL stations (NRCS 2017), while those from Toolik Lake are from the Toolik Field Station meteorological tower (EDCT 2017)

Month	June	July	August	June	July	August
Parameter	Coldfoot: 2002–2016			Coldfoot: 2016		
*T*_mean_	14.3 ± 0.2	14.2 ± 0.2	10.9 ± 0.2	13.0 ± 0.6	15.2 ± 0.6	12.2 ± 0.4
*T*_min_	7.1 ± 0.1	8.1 ± 0.1	5.1 ± 0.2	5.9 ± 0.5	9.0 ± 0.6	7.1 ± 0.6
*T*_max_	21.0 ± 0.2	20.5 ± 0.2	17.2 ± 0.2	19.6 ± 0.9	21.6 ± 0.8	18.0 ± 0.5

	Toolik Lake: 2002–2016			Toolik Lake: 2016		
*T*_mean_	8.8 ± 0.2	10.8 ± 0.2	7.3 ± 0.2	6.5 ± 1.2	11.3 ± 0.8	8.5 ± 0.7
*T*_min_	3.1 ± 0.2	5.1 ± 0.2	2.1 ± 0.2	1.2 ± 1.0	5.2 ± 0.7	3.5 ± 0.5
*T*_max_	13.8 ± 0.3	15.4 ± 0.2	12.1 ± 0.3	11.9 ± 1.3	16.2 ± 1.0	13.1 ± 0.8

	Sagwon: 2002–2016			Sagwon: 2016		
*T*_mean_	8.2 ± 0.2	10.6 ± 0.2	7.7 ± 0.2	7.7 ± 1.0	11.4 ± 0.9	8.3 ± 0.8
*T*_min_	3.1 ± 0.2	5.7 ± 0.2	3.8 ± 0.2	3.0 ± 0.8	6.5 ± 0.8	4.2 ± 0.6
*T*_max_	13.3 ± 0.3	15.4 ± 0.2	11.8 ± 0.3	12.6 ± 1.2	16.5 ± 1.0	12.8 ± 0.9

### 3.2 Characterization of ambient and OTC growing environments

During the 2016 growing season, PAR data from the TL garden indicated that light availability was consistently lower in the OTC treatment (data not shown). For the nine measured days in June, the percent difference in PAR between treatments was 25%, while it was 16% and 9% in July and August, respectively. These values represent hours of the day when PAR was >500 μmol m^−2^ s^−1^ for at least one of the treatments.

Over a 24‐hr period, mean *T*_air_ in the OTCs was higher than in the ambient treatment at both the TL and SAG gardens, although the degree of warming was greater at TL (Table 2). The OTC treatments at TL were 1.0–1.9°C warmer than ambient treatments depending on the month, while at SAG, OTCs produced temperatures 0.3–0.8°C warmer than ambient (Table 2). The OTC treatment was likely less effective at the SAG garden because the site is windier than TL (J. L. Schedlbauer, personal observation).

**Table 2:** Comparison of temperature environments between ambient and open‐top chamber (OTC) treatments at the Toolik Lake and Sagwon transplant gardens in June, July, and August 2016. Mean ± *SE* air temperature (*T*_air_) data for 24 hr are shown, as well as mean temperature differences between treatments

	24‐hr Mean *T*_air_ (°C)		
	Ambient	OTC	Difference
Toolik Lake			
June	9.2 ± 0.30	11.0 ± 0.35	1.8
July	12.1 ± 0.25	14.0 ± 0.30	1.9
August	8.6 ± 0.22	9.6 ± 0.25	1.0
Sagwon			
June	9.9 ± 0.29	10.7 ± 0.32	0.8
July	12.8 ± 0.26	13.4 ± 0.26	0.6
August	8.7 ± 0.23	9.1 ± 0.24	0.3

Given that the TL and SAG gardens did not differ dramatically in growing season temperature and the degree of warming provided by the OTC was lower at the SAG garden, relative to the TL garden, the data collected in this study reflect a gradient of warming. Specifically, the TL and SAG garden ambient treatments were nearly identical (SAG was slightly warmer), moderate warming was provided by the SAG OTC treatment, and the greatest warming was provided by the TL‐OTC treatment.

### 3.3 Leaf morphology and leaf nitrogen content

Within both the TL and SAG transplant gardens, SLA did not vary by ecotype or treatment, and there was no interaction between the main effects (*p* > .05, Table 3). Specific leaf area varied between 91.71 and 103.96 cm^2^/g (Table 3). Similarly, N_area_ was invariant across ecotypes and treatments at each garden, and no ecotype by treatment interaction was detected (*p* > .05, Table 3). N_area_ ranged from 1.59 to 1.90 g N/m^2^ (Table 3).

**Table 3:** Mean ± *SE* specific leaf area (SLA) and leaf nitrogen content per unit leaf area (N_area_) for *Eriophorum vaginatum* foliage. No significant differences in SLA or N_area_ were found among ecotypes (CF—Coldfoot, TL—Toolik Lake, SAG—Sagwon) or between treatments (ambient or OTC—open‐top chamber) within either garden (*p* > .05)

		SLA (cm^2^/g)		N_area_ (g N/m^2^)	
Ecotype	Treatment	Toolik Lake Garden	Sagwon Garden	Toolik Lake Garden	Sagwon Garden
CF	Ambient	100.37 ± 3.06	103.96 ± 3.59	1.90 ± 0.09	1.59 ± 0.09
	OTC	97.19 ± 2.83	96.44 ± 4.44	1.88 ± 0.10	1.66 ± 0.07
TL	Ambient	95.74 ± 1.78	96.28 ± 2.60	1.88 ± 0.11	1.88 ± 0.11
	OTC	99.39 ± 4.28	94.00 ± 2.88	1.81 ± 0.06	1.72 ± 0.10
SAG	Ambient	91.85 ± 3.34	91.71 ± 2.69	1.74 ± 0.08	1.87 ± 0.10
	OTC	96.80 ± 1.40	93.81 ± 4.08	1.72 ± 0.08	1.64 ± 0.05

### 3.4 Temperature response of *V*_cmax_, *J*_max_, *A*_net_, and *R*_d_

**Figure 3:**
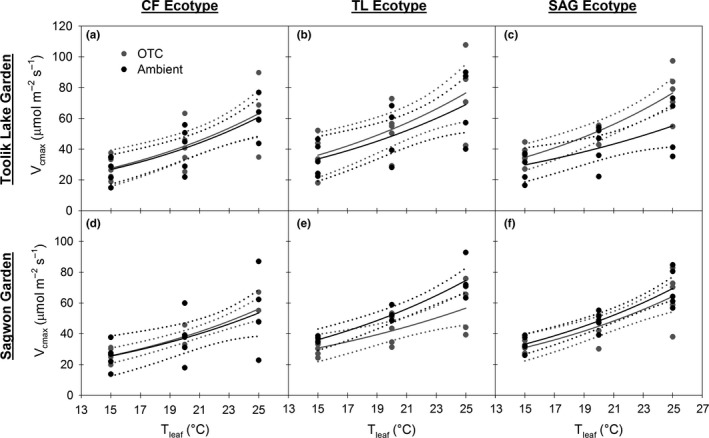
Maximum carboxylation rate of Rubisco (*V*_cmax_) versus leaf temperature (*T*_leaf_) for each ecotype of *Eriophorum vaginatum* (CF—Coldfoot, TL—Toolik Lake, SAG—Sagwon) within each garden (a–f). Each panel shows data and models for both the ambient (black) and open‐top chamber (OTC) treatments (gray). Solid lines show the Arrhenius function used to model these relationships; dotted lines are 95% confidence intervals

**Figure 4:**
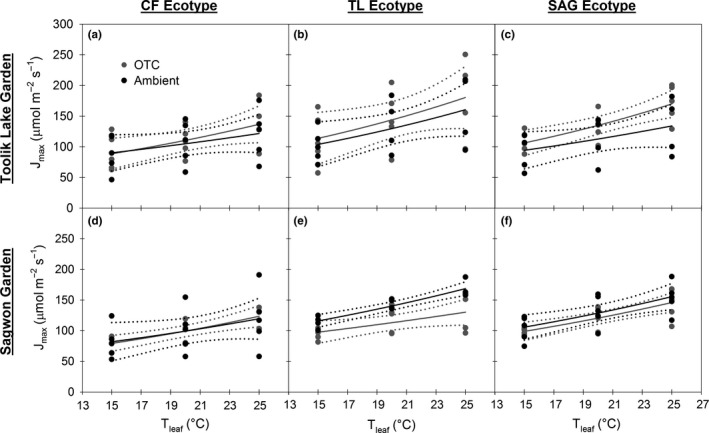
Maximum rate of electron transport (*J*_max_) versus leaf temperature (*T*_leaf_) for each ecotype of *Eriophorum vaginatum* (CF—Coldfoot, TL—Toolik Lake, SAG—Sagwon) within each garden (a–f). Each panel shows data and models for both the ambient (black) and open‐top chamber (OTC) treatments (gray). Solid lines show the Arrhenius function used to model these relationships; dotted lines are 95% confidence intervals

At the TL garden, treatment did not differentially affect the relationship between *V*_cmax_ or *J*_max_ and temperature for the CF and TL ecotypes (Figures 3a,b, and 4a,b). However, for the SAG ecotype, the OTC treatment had higher *V*_cmax_ and *J*_max_ values at measurement temperatures of 20 and 25°C, as there was little overlap in the models’ 95% CIs (Figures 3c and 4c). Accordingly, the OTC treatment had somewhat higher values for *E*_a_, a term that reflects the exponential rate of increase in enzyme activity with temperature (Table 4). At the SAG garden, both the CF and SAG ecotypes had similar *V*_cmax_ versus *T*_leaf_ and *J*_max_ versus *T*_leaf_ relationships, regardless of treatment (Figures 3d,f and 4d,f), while the TL ecotype exhibited different responses by treatment (Figures 3e and 4e). Specifically, *V*_cmax_ and *J*_max_ in the ambient treatment were higher at temperatures of 20 and 25°C (Figures 3e and 4e). Use of the Arrhenius function to model *V*_cmax_ or *J*_max_ in relation to measurement temperature generally yielded significant relationships (*p* < .05) with model *SE* <30 (in 19 or 24 cases, Table 4). Overall, the Arrhenius function performed better when modeling *V*_cmax_ than *J*_max_, as there was higher variation in the *J*_max_ data (Figures 3 and 4, Table 4).

**Table 4:** Outputs of an Arrhenius function used to model the maximum carboxylation rate of Rubisco (*V*_cmax_) and maximum rate of electron transport (*J*_max_) versus leaf temperature for each ecotype of *Eriophorum vaginatum* (CF—Coldfoot, TL—Toolik Lake, SAG—Sagwon) and treatment (ambient or OTC—open‐top chamber) within each transplant garden. Parameter estimates ±*SE* include *V*_cmax_ and *J*_max_ at 25°C (*V*_cmax_(25) and *J*_max_(25), respectively), as well as *E*_a_, the activation energy for either *V*_cmax_ or *J*_max_

Ecotype	Treatment	*V*_cmax_(25) (μmol m^−2^ s^−1^)	*E*_a_ (kJ/mol)	Model *SE*	*R* ^2^_adj_	*F*	*p*
Toolik Lake Garden							
CF	Ambient	60.8 ± 5.7	56.8 ± 14.2	11.9	.57	18.2	.0011
	OTC	63.1 ± 7.0	57.3 ± 16.9	14.7	.48	13.0	.0036
TL	Ambient	68.9 ± 8.2	49.6 ± 17.1	17.2	.41	9.4	.0106
	OTC	76.5 ± 8.6	52.1 ± 16.4	18.0	.44	11.2	.0058
SAG	Ambient	55.0 ± 6.3	42.7 ± 15.6	13.3	.36	8.2	.0142
	OTC	76.5 ± 4.5	54.9 ± 9.5	10.4	.75	40.0	<.0001
Sagwon Garden							
CF	Ambient	53.5 ± 7.0	51.3 ± 20.2	16.3	.32	7.5	.0171
	OTC	56.1 ± 3.3	54.0 ± 8.1	6.1	.79	46.5	<.0001
TL	Ambient	74.7 ± 3.6	50.2 ± 7.4	7.5	.83	53.1	<.0001
	OTC	56.6 ± 5.0	42.4 ± 12.1	10.7	.48	13.2	.0034
SAG	Ambient	69.4 ± 3.5	51.0 ± 7.8	8.2	.78	49.6	<.0001
	OTC	64.2 ± 4.6	50.5 ± 11.0	10.8	.62	23.8	.0003

Ecotype	Treatment	*J*_max_(25) (μmol m^−2^ s^−1^)	*E*_a_ (kJ/mol)	Model *SE*	*R* ^2^_adj_	*F*	*p*
Toolik Lake Garden							
CF	Ambient	121.5 ± 14.5	20.8 ± 14.6	34.6	.07	2.1	.1705
	OTC	136.6 ± 13.8	30.0 ± 12.6	29.6	.27	5.8	.0328
TL	Ambient	160.3 ± 19.6	30.1 ± 15.1	41.7	.21	4.2	.0662
	OTC	180.2 ± 23.3	32.0 ± 16.3	49.9	.19	4.0	.0691
SAG	Ambient	133.9 ± 16.3	24.4 ± 14.6	35.2	.13	2.9	.1139
	OTC	169.9 ± 10.2	32.5 ± 8.0	24.0	.56	17.5	.0013
Sagwon Garden							
CF	Ambient	119.6 ± 15.5	26.1 ± 16.5	36.9	.10	2.6	.1295
	OTC	123.6 ± 7.9	30.7 ± 7.5	15.0	.56	16.5	.0019
TL	Ambient	168.7 ± 5.0	26.0 ± 3.8	10.6	.82	50.0	<.0001
	OTC	130.3 ± 9.7	19.9 ± 8.7	21.1	.26	5.5	.0377
SAG	Ambient	155.6 ± 9.9	26.7 ± 8.1	23.6	.43	11.4	.0050
	OTC	146.0 ± 7.4	26.9 ± 6.5	17.6	.55	18.2	.0009

**Figure 5:**
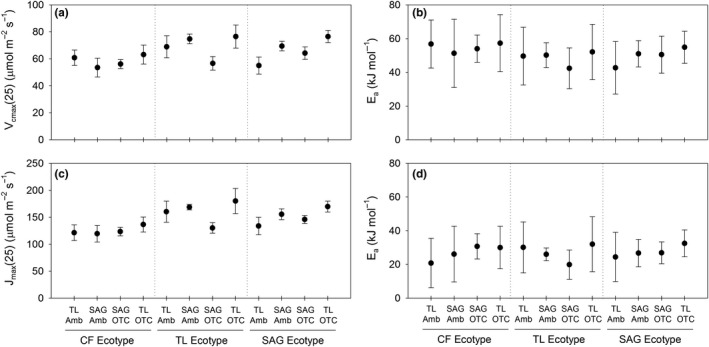
Plots of parameter estimates ± *SE* for the Arrhenius functions used to model the maximum carboxylation rate of Rubisco (*V*_cmax_) and maximum rate of electron transport (*J*_max_) versus leaf temperature for each ecotype of *Eriophorum vaginatum* (CF—Coldfoot, TL—Toolik Lake, SAG—Sagwon) and treatment (ambient—Amb or open‐top chamber—OTC) within each transplant garden. Parameter estimates are arranged within each ecotype along the temperature gradient provided by treatments, where TL ambient was the coolest treatment and TL‐OTC was the warmest treatment. Shown are estimates of (a) *V*_cmax_ at 25°C (*V*_cmax_(25)), (b) activation energy (*E*_a_) for *V*_cmax_, (c) *J*_max_ at 25°C (*J*_max_(25)), and (d) *E*_a_ for *J*_max_

**Figure 6:**
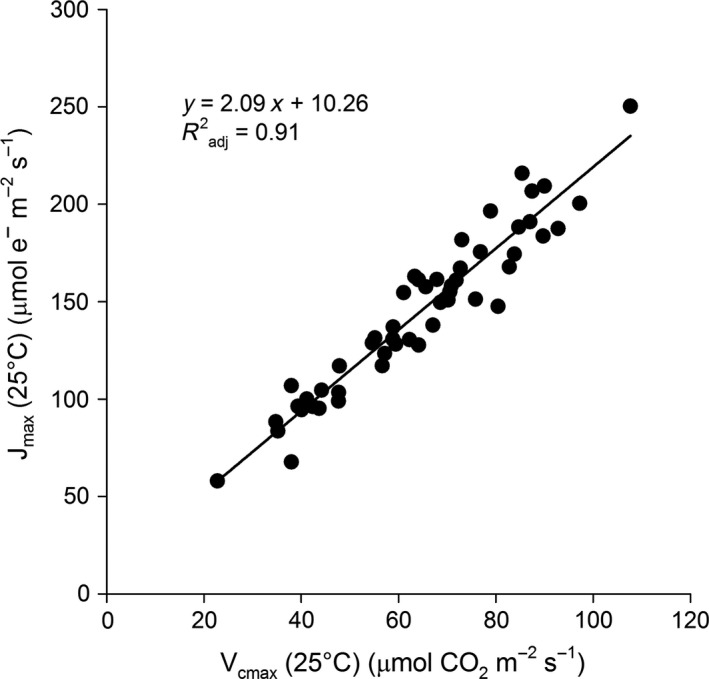
Linear regression of the maximum rate of electron transport at 25°C (*J*_max_(25)) against the maximum carboxylation rate of Rubisco at 25°C (*V*_cmax_(25)) for *E. vaginatum*. Because the ratio of these terms did not vary significantly by garden, ecotype, or treatment (*p* > .05), all data were pooled for this analysis

Given the gradient of warming provided by treatments in this study (see above), estimates of the parameters *V*_cmax_(25), *J*_max_(25), and *E*_a_ were examined in relation to this gradient. The CF and TL ecotypes did not exhibit strong variation in *V*_cmax_(25) or *J*_max_(25) with increased growth temperature, as evidenced by a high degree of overlap in the *SE* of these parameter estimates (Figure 5a,c, Table 4). However, the SAG ecotype exhibited increases in both parameters with increased growth temperature, such that both parameters were much higher in the TL garden OTC treatment than in the TL‐ambient treatment (Figure 5a,c, Table 4). When examining just the warmest treatment (i.e., TL‐OTC), estimates of *V*_cmax_ and *J*_max_ were higher for the TL and SAG ecotypes, relative to the CF ecotype (Figure 5a,c, Table 4). A plot of *J*_max_(25) versus *V*_cmax_(25) for each measured tussock yielded a positive linear relationship between the two variables, with a slope of 2.09 (*R* ^2^_adj_ = .91, Figure 6). Estimates of *E*_a_ for both *V*_cmax_ and *J*_max_ were largely invariant within and across ecotypes because of the magnitude of the standard errors associated with these parameter estimates (Figure 5b,d, Table 4).

**Figure 7:**
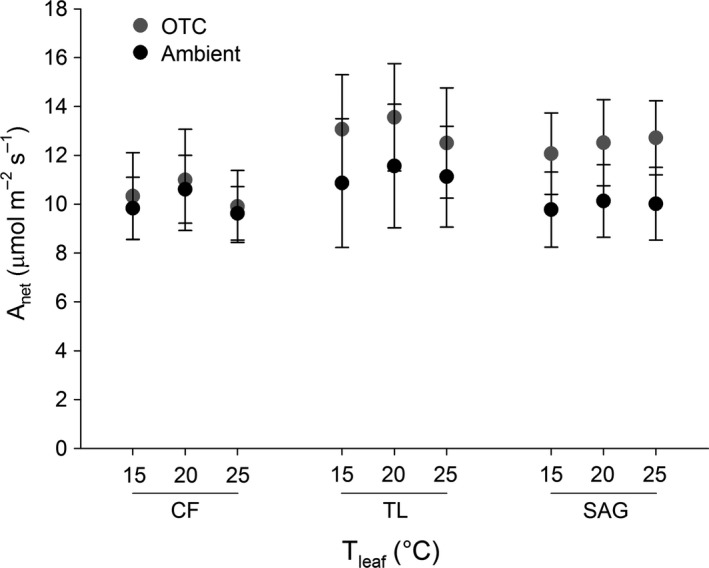
Mean ± *SE* net photosynthesis (*A*_net_) for each *E. vaginatum* ecotype (CF—Coldfoot, TL—Toolik Lake, SAG—Sagwon) and leaf measurement temperature (*T*_leaf_). Data are shown for the Toolik Lake garden only, as the treatment effect (i.e., degree of warming) was most effective at this site. Separate ANOVAs for each ecotype indicated no significant differences in *A*_net_ with treatment or *T*_leaf_ (*p* > .05)

Despite moderate differences in temperature between treatments at the TL garden (Table 2), *A*_net_ did not vary significantly for any of the ecotypes with treatment or measurement temperature (*p* > .05, Figure 7, Table 5). However, the patterns reported above for *V*_cmax_(25) and *J*_max_(25) are reflected in mean *A*_net_ values, such that the OTC treatment tended to produce marginally higher *A*_net_ in TL and SAG ecotypes across measurement temperatures. This pattern was particularly notable for the SAG ecotype (*p* = .068) and contrasts with nearly identical mean *A*_net_ values across treatments for the CF ecotype (*p* = .763). Rates of *A*_net_ ranged from 9.6 to 13.6 μmol m^−2^ s^−1^ (Figure 7).

**Table 5:** Results of two‐way ANOVAs run separately for each ecotype (CF—Coldfoot, TL—Toolik Lake, SAG—Sagwon) at the TL transplant garden. Treatment, leaf temperature (*T*_leaf_), and their interaction were analyzed to determine their effect on *Eriophorum vaginatum* net photosynthesis (*A*_net_)

		Toolik Lake Garden	
Ecotype	Main effect	*F*‐value	*p*
CF	Treatment	0.0932	.7628
	*T*_leaf_	0.2360	.7916
	Treatment × *T*_leaf_	0.0024	.9976
TL	Treatment	1.7947	.1929
	*T*_leaf_	0.0766	.9265
	Treatment × *T*_leaf_	0.0754	.9276
SAG	Treatment	3.6488	.0681
	*T*_leaf_	0.0479	.9533
	Treatment × *T*_leaf_	0.0090	.9911

**Figure 8:**
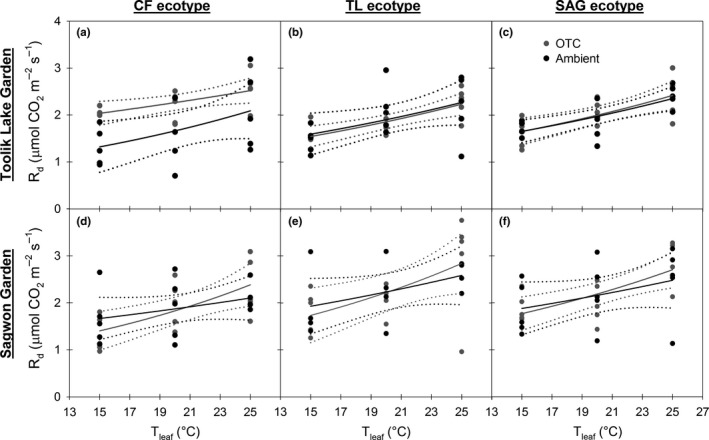
Dark respiration (*R*_d_) versus leaf temperature (*T*_leaf_) for each ecotype of *Eriophorum vaginatum* (CF—Coldfoot, TL—Toolik Lake, SAG—Sagwon) within each garden (a–f). Each panel shows data and models for both the ambient (black) and open‐top chamber treatments (gray). Solid lines show the exponential function used to model these relationships and dotted lines are 95% confidence intervals

In evaluating the relationship between *R*_d_ and temperature, all but one comparison yielded comparable model fits between treatments within each garden (Figure 8a–f). The exception was for the CF ecotype at the TL garden, where the OTC treatment had higher rates of *R*_d_ across all measurement temperatures, relative to the ambient treatment (Figure 8a). The *Q*_10_ of *R*_d_ computed for each curve ranged from 1.23 to 1.70 (Table 6). Examining *Q*_10_ values for each ecotype along the gradient of warming described above did not yield any positive or negative trends (Table 6). The exponential function used to model the *R*_d_ versus *T*_leaf_ relationship yielded significant relationships (*p* < .05) in seven of 12 cases, but overall model fits were of poor quality (i.e., model *SE* > 30 and *R* ^2^_adj_ < .40 in the majority of cases) given high variance in the data (Figure 8, Table 6).

**Table 6:** Outputs of an exponential function used to model dark respiration (*R*_d_) versus leaf temperature for each ecotype (CF—Coldfoot, TL—Toolik Lake, SAG—Sagwon) of *E. vaginatum* and treatment (ambient or OTC—open‐top chamber) within each transplant garden. Also included is the computed *Q*_10_ value for each model that was fit

Ecotype	Treatment	*Q*_10_	Model *SE*	*R* ^2^_adj_	*F*	*p*
Toolik Lake Garden						
CF	Ambient	1.58	0.65	.15	3.5100	.0837
	OTC	1.23	0.30	.28	6.5668	.0236
TL	Ambient	1.42	0.53	.19	4.3130	.0582
	OTC	1.45	0.26	.53	16.7464	.0013
SAG	Ambient	1.42	0.29	.49	14.4374	.0022
	OTC	1.46	0.34	.47	13.2927	.0030
Sagwon Garden						
CF	Ambient	1.26	0.52	.05	1.7321	.2109
	OTC	1.70	0.50	.39	10.0976	.0073
TL	Ambient	1.34	0.60	.11	2.3898	.1532
	OTC	1.63	0.70	.27	6.0926	.0282
SAG	Ambient	1.32	0.66	.07	2.1001	.1710
	OTC	1.52	0.42	.43	11.6807	.0046

### 3.5 Relationship between *g*_s_ and *D*

**Figure 9:**
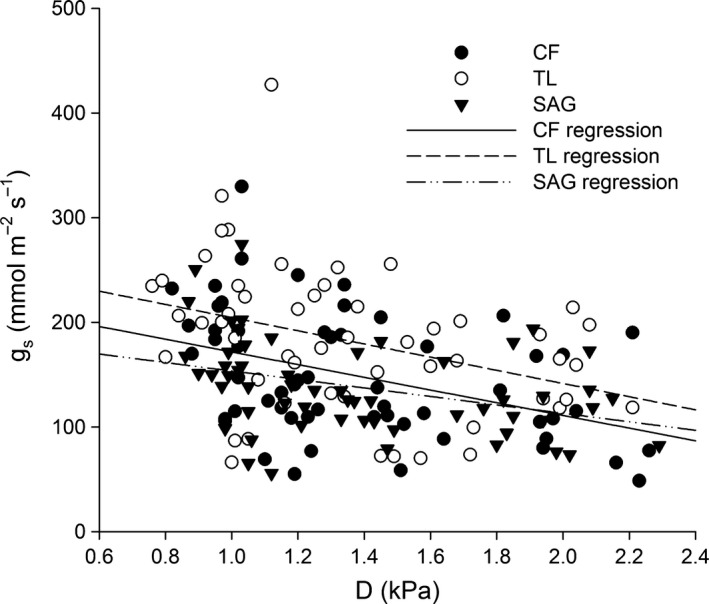
Linear regressions showing the relationship between stomatal conductance (*g*_s_) and vapor pressure deficit (*D*) for each ecotype of *Eriophorum vaginatum* (CF—Coldfoot, TL—Toolik Lake, SAG—Sagwon). Data within each ecotype were pooled across treatments and gardens and were extracted from gas exchange measurements made at 400 ppm CO_2_. The equation for the CF ecotype (solid black circles, solid black line) was *y* = −60.67*x* + 232, *R* ^2^_adj_ = .15, for the TL ecotype (open circles, dashed line) was *y* = −62.97*x* + 267, *R* ^2^_adj_ = .11, and for the SAG ecotype (black triangles, dash‐dot line) was *y* = −40.48*x* + 194, *R* ^2^_adj_ = .12

Negative relationships between *g*_s_ and *D* were found for all three ecotypes, although only 11%–15% of the variation in the *g*_s_ was explained by *D* (Figure 9). For CF and TL ecotypes, linear regression revealed similar slopes (−60.67 and −62.97, respectively), while the SAG ecotype had a shallower slope (−40.48), indicating less responsiveness of *g*_s_ to changes in *D* (Figure 9). Of the measurements made when *D* was >1.5 kPa, 91% were made at a *T*_leaf_ of 25°C.

## 4 DISCUSSION

### 4.1 Photosynthetic temperature responses

Contrary to expectations of detractive adjustments in photosynthesis for *E. vaginatum* given increased growth temperature, there was evidence of enhanced or no change in photosynthetic capacity with higher growth temperature. Enhancements were most pronounced for the northern ecotype (SAG), with substantial increases in both *V*_cmax_(25) and *J*_max_(25), between the coolest and warmest growing conditions. The SAG ecotype also had a marginally significant increase in *A*_net_ with increased growth temperature. The most southern ecotype, CF, displayed evidence of photosynthetic homeostasis across growth temperatures, both in terms of photosynthetic capacity and *A*_net_. Responses of the mid‐latitude TL ecotype to increased growth temperature fell between that of the northern and southern ecotypes. These findings suggest that ecotypic specialization affects photosynthetic responses to growth temperature, although not in the ways initially expected. Over the range of warming provided in this experiment, there was no evidence of a photosynthetic underpinning to adaptational lag in *E. vaginatum*.

In many ways, photosynthetic responses to the treatments imposed in the present study showed a graduated response to increased temperature as a function of an ecotype's latitude of origin. While the range of temperatures to which tussocks were exposed was likely within or slightly below the historic range of variation for the CF ecotype, the OTC treatments provided warmer than average conditions for both the TL and SAG ecotypes. Although mean growing season temperatures over the past 15 years were similar between the Toolik Lake and Sagwon sites, photosynthetic data suggest that the SAG *E. vaginatum* ecotype is adapted to a local climate that has historically been different (possibly cooler and/or more variable) than at Toolik Lake. Unfortunately, historical temperature data from Alaska's north slope are limited to 2–4 years of data from the 1970s, and they provide an insufficient baseline for the region's climate given the short time period and high interannual variation (Haugen, 1982).

The shift in photosynthetic responses to increased growth temperature from homeostasis in the south to temperature acclimation in the north must be interpreted in light of the south to north decline in growing season temperature coupled with the degree of warming provided by the OTC treatments. In context, it is probable that photosynthetic enhancements with increased growth temperature exist for all three ecotypes given a moderate (at least up to ~2°C) temperature increase. However, the methodological limitations of the present study made it impossible to assess the effect of warming above historical conditions on the CF ecotype. Evidence of photosynthetic acclimation to increased growth temperature is consistent with much of the literature, although it was unexpected for a species adapted to a relatively cool climate (Berry & Björkman, 1980; Kattge & Knorr, 2007; Way & Yamori, 2014; Yamori et al., 2014). If the SAG *E. vaginatum* ecotype in the present study serves as a model for the species when exposed to moderately elevated growth temperature, relative to historical conditions, modest enhancements in carbon uptake should be expected. Certainly, further research is required to determine whether this is the case. It should also be noted that a moderate decline in *g*_s_ with increased *D* was found for all *E. vaginatum* ecotypes. This indicates that photosynthesis may have been limited at a measurement temperature of 25°C, thereby masking additional photosynthetic enhancement. However, because little of the variation in *g*_s_ was explained by *D* across ecotypes (*R* ^2^_adj_ = .11–.15), the effect is likely to be minor.

Potential enhancements in carbon gain contrast with the lower population growth rates observed for northern ecotypes transplanted into warmer, southern gardens (Chandler et al., 2015; McGraw et al., 2015). Additionally, Heskel et al. (2013) found that an increase in growth temperature of 5°C negatively affected *A*_net_ for the TL *E. vaginatum* ecotype. Further, Souther et al. (2014) compiled data from six reciprocally transplanted *E. vaginatum* ecotypes and found that *A*_net_ was maximized when TDD was lower (i.e., growing conditions were cooler) than at the home‐site, thereby suggesting the negative effects of a warming climate on *E. vaginatum*. These contrasting findings raise two possibilities: (1) Ecotypes of *E. vaginatum* do not allocate fixed carbon from enhanced photosynthesis to aboveground growth. Instead, respiratory costs, root or rhizome growth, and storage are potential avenues of carbon allocation; (2) The degree of warming provided by the present study was within the tolerance limits of photosynthesis for these *E. vaginatum* ecotypes, but warmer temperatures do lead to negative effects on growth. These possibilities can be investigated in part by considering findings related to the *R*_d_ growth temperature response.

### 4.2 Respiratory temperature responses

Respiratory responses to increased growth temperature typically show evidence of thermal acclimation, such that warm‐grown plants have lower respiration rates than cold‐grown plants when measured at the same temperature, and these responses have previously been observed for *E. vaginatum* (Heskel et al., 2014; Kornfeld et al., 2013; Souther et al., 2014; van de Weg et al., 2013). Acclimation can result from either a decreased *Q*_10_ (i.e., Type I acclimation—lower temperature responsiveness at high temperature) or a decrease in *R*_d_ at both low and high temperature (i.e., Type II acclimation), and both of these responses are thought to be driven by substrate or adenylate availability (Atkin & Tjoelker, 2003). The present study shows that, with the exception of the CF ecotype grown at the TL garden, responses of *R*_d_ to increased measurement temperature did not differ substantially between ambient and OTC treatments. In the case of the CF ecotype at the TL garden, *R*_d_ was lower under ambient conditions, relative to the OTC treatment. Because TL‐ambient conditions were cooler than typical for the CF ecotype, the decrease in *R*_d_ may represent an enzymatic limitation not experienced by the more cold‐adapted TL and SAG ecotypes.

Overall, there was no evidence of thermal acclimation in *R*_d_ given the growth temperatures provided by this experiment. Because photosynthetic capacity was not negatively affected by increased growth temperature, respiratory substrate and adenylate availability likely remained high enough to maintain *R*_d_ across treatments. However, multiple reports of respiratory acclimation to increased growth temperature in *E. vaginatum* (Heskel et al., 2014; Kornfeld et al., 2013; Souther et al., 2014) raises the likelihood that a sustained growth temperature 4–5°C warmer than ambient (as in Kornfeld et al., 2013; Heskel et al., 2014) is an important threshold for *E. vaginatum* that leads to physiological adjustment. These conclusions are consistent with the second hypothesis described above.

Across ecotypes, there was a high degree of overlap in modeled responses of *R*_d_ to measurement temperature, as well as *Q*_10_ values, suggesting little ecotypic variation in *R*_d_. These uniform responses are unlikely to differentially affect overall tussock carbon balance for different ecotypes. The *Q*_10_ values for *E. vaginatum* across ecotypes and treatments were exceptionally low (1.23–1.70) relative to typical values for Arctic plants (2.56, Atkin & Tjoelker, 2003). This suggests that *E. vaginatum* is less responsive to increased growth temperature than many Arctic plants, an attribute likely to be beneficial in terms of plant carbon balance as Arctic temperatures continue to rise.

### 4.3 Photosynthetic capacity of *E. vaginatum* in context

While *E. vaginatum V*_cmax_(25) and *J*_max_(25), as well as *E*_a_ of *V*_cmax_ were within the range of values reported across functional types comprised of both temperate and Arctic plants (i.e., crops, deciduous tree, coniferous trees, herbs, grasses, sedges, deciduous shrubs), the *E*_a_ of *J*_max_ was at the lower end of reported values (Kattge & Knorr, 2007; Medlyn et al., 2002; Rogers, Serbin, Ely, Sloan, & Wullschleger, 2017). *Eriophorum vaginatum*'s consistent ratio of *J*_max_(25) to *V*_cmax_(25), regardless of garden, ecotype, or treatment, indicated that *J*_max_ and *V*_cmax_ were similarly responsive to changes in growth temperature. Onoda, Hikosaka, and Hirose (2005) suggested that species without *J*_max_/*V*_cmax_ plasticity in response to growth temperature may have photosynthetic rates that are limited by Rubisco carboxylation (*V*_cmax_) over the range of growth temperatures. Additionally, the slope of the relationship between *J*_max_(25) and *V*_cmax_(25) for *E. vaginatum* was 2.09, a value somewhat greater than those reported in synthesis studies focused principally on temperate species (slopes of 1.67 and 1.89, Medlyn et al., 2002; Kattge & Knorr, 2007). Recent work on seven species from Alaskan coastal tundra yielded a mean slope of 2.53, indicating that Arctic species likely maintain a higher ratio than temperate species (Rogers, Serbin et al., 2017).

Differences between temperate and Arctic plants show that Arctic species, including *E. vaginatum*, allocate more resources to light harvesting and electron transport, relative to temperate species (Rogers, Serbin et al., 2017). This may be an adaptation to life in the relatively low‐light growing conditions of the Arctic. Further, a high *J*_max_(25)/*V*_cmax_(25) should confer a physiological advantage under elevated CO_2_ because ribulose‐1,5‐bisphosphate (RuBP) regeneration will not limit A over a relatively wide range of *C*_i_ (Rogers, Medlyn et al., 2017; Rogers, Serbin et al., 2017). Photosynthetic rates of Arctic plants should therefore experience high responsiveness to rising CO_2_, although further study is required to assess the magnitude of elevated *J*_max_/*V*_cmax_ in the Arctic. As noted by Medlyn et al. (2002) and Rogers, Serbin et al. (2017), additional research is needed to develop a more robust understanding of photosynthetic capacity in plants from cold climates. Such data are of key importance, as Rogers, Serbin et al. (2017) recently demonstrated that terrestrial biosphere models underestimate Arctic plant photosynthetic capacity, largely due to lack of data.

### 4.4 Leaf morphology and nitrogen content

In addition to physiology, growth temperature often affects leaf morphology, such that plants grown under cooler temperature regimes have lower SLA, which may enhance photosynthesis, as it is often coupled with higher N content (Campbell et al., 2007; Yamori et al., 2005). Further, because approximately half a leaf's N content is associated with the photosynthetic apparatus (Evans, 1989; Evans & Seemann, 1989), an assessment of variation in N_area_ among ecotypes and treatments may explain some of the observed variation in photosynthetic capacity. Both SLA and N_area_ were invariant for *E. vaginatum* among ecotypes and between treatments. These findings indicate that any photosynthetic responses to growth temperature within each garden were not related to underlying variation in leaf morphology. However, the maintenance of low‐temperature enzyme capacity, as indicated by constant N_area_ with increased growth temperature, should support enhanced photosynthetic capacity and *A*_net_, although this response was only observed for the SAG ecotype. Overall, the N_area_ analysis suggests a lack of plasticity in enzyme concentration among the three ecotypes of *E. vaginatum*, and further research is merited to determine how leaf N affects photosynthetic capacity in this species.

## 5 CONCLUSIONS

Over the range of growth temperatures examined in the present study, no evidence of a physiological underpinning to adaptational lag was observed for *E. vaginatum*. Instead of detractive adjustments in photosynthesis with increased growth temperature, photosynthetic homeostasis or enhancement was observed, depending on an ecotype's latitude of origin. Contrary to expectations, the greatest enhancement in photosynthetic capacity and rate occurred in the northernmost ecotype. Further, no evidence of temperature acclimation was observed in measurements of *R*_d_, and all ecotypes responded similarly to variation in growth temperature. Together, these findings suggest that moderate enhancements in growth temperature (up to ~2°C) do not negatively affect the physiological performance of *E. vaginatum*. Despite neutral to modest enhancements in photosynthesis given moderate warming, this appears to confer no lasting advantage to *E. vaginatum* as growth temperature rises. Other experimental warming treatments indicate that warming of ~5°C above ambient over the course of 20 years is enough to shift the species composition of Alaska's tussock tundra toward a more shrub‐dominated ecosystem (Heskel et al., 2013), a pattern that has been documented throughout the rapidly warming Arctic (Tape, Sturm, & Racine, 2006). Within the moist tussock tundra, it is likely that *E. vaginatum* will eventually be unable to effectively compete with shrubs, although the present study shows there remains a window of time before negative physiological effects are evident.

## CONFLICT OF INTEREST

None declared.

## AUTHOR CONTRIBUTIONS

JLS designed and carried out the research, data analysis, and data interpretation, drafted the manuscript, and approved the final manuscript. NF conceived of the experimental design that made this study possible, maintained transplant gardens, generated the underlying idea for this study, aided in developing methods, provided comments on the manuscript, and approved the final manuscript. KH contributed substantially to data collection, provided comments on the manuscript, and approved the final manuscript. MLM and JT conceived of the experimental design that made this study possible, maintained transplant gardens, provided comments on the manuscript, and approved the final manuscript.

## References

[ece33939-bib-0001] ACIA [Arctic Climate Impact Assessment] (2004). Impacts of a warming Arctic. Cambridge, UK: Cambridge University Press.

[ece33939-bib-0002] Aitken, S. N. , Yeaman, S. , Holliday, J. A. , Wang, T. , & Curtis‐McLane, S. (2008). Adaptation, migration or extirpation: Climate change outcomes for tree populations. Evolutionary Applications, 1, 95–111. https://doi.org/10.1111/j.1752-4571.2007.00013.x 25567494 10.1111/j.1752-4571.2007.00013.x PMC3352395

[ece33939-bib-0003] Anderson, J. T. , Panett, A. M. , & Mitchell‐Olds, T. (2012). Evolutionary and ecological responses to anthropogenic climate change. Plant Physiology, 160, 1728–1740. https://doi.org/10.1104/pp.112.206219 23043078 10.1104/pp.112.206219 PMC3510106

[ece33939-bib-0004] Atkin, O. K. , Bruhn, D. , & Tjoelker, M. G. (2005). Response of plant respiration to changes in temperature: Mechanisms and consequences of variations in Q_10_ values and acclimation In Lambers H., & Ribas‐Carbo M. (Eds.), Plant respiration: From cell to ecosystem (pp. 95–135). Dordrecht, the Netherlands: Springer https://doi.org/10.1007/1-4020-3589-6

[ece33939-bib-0005] Atkin, O. K. , & Tjoelker, M. G. (2003). Thermal acclimation and the dynamic response of plant respiration to temperature. Trends in Plant Science, 8, 343–351. https://doi.org/10.1016/S1360-1385(03)00136-5 12878019 10.1016/S1360-1385(03)00136-5

[ece33939-bib-0006] Bennington, C. C. , Fetcher, N. , Vavrek, M. C. , Shaver, G. R. , Cummings, K. J. , & McGraw, J. B. (2012). Home site advantage in two long‐lived arctic plant species: Results from two 30‐year reciprocal transplant studies. Journal of Ecology, 100, 841–851. https://doi.org/10.1111/j.1365-2745.2012.01984.x

[ece33939-bib-0007] Bernacchi, C. J. , Singsaas, E. L. , Pimentel, C. , Portis, A. R. Jr , & Long, S. P. (2001). Improved temperature response functions for models of Rubisco‐limited photosynthesis. Plant, Cell and Environment, 24, 253–259. https://doi.org/10.1111/j.1365-3040.2001.00668.x

[ece33939-bib-0008] Berry, J. , & Björkman, O. (1980). Photosynthetic response and adaptation to temperature in higher plants. Annual Review of Plant Physiology, 31, 491–543. https://doi.org/10.1146/annurev.pp.31.060180.002423

[ece33939-bib-0009] Campbell, C. , Atkinson, L. , Zaragoza‐Castells, J. , Lundmark, M. , Atkin, O. , & Hurry, V. (2007). Acclimation of photosynthesis and respiration is asynchronous in response to changes in temperature regardless of plant functional group. New Phytologist, 176, 375–389. https://doi.org/10.1111/j.1469-8137.2007.02183.x 17692077 10.1111/j.1469-8137.2007.02183.x

[ece33939-bib-0010] Chandler, J. L. , McGraw, J. B. , Bennington, C. , Shaver, G. R. , Vavrek, M. C. , & Fetcher, N. (2015). Tiller population dynamics of reciprocally transplanted *Eriophorum vaginatum* L. ecotypes in a changing climate. Population Ecology, 57, 117–126. https://doi.org/10.1007/s10144-014-0459-9

[ece33939-bib-0011] Chapin, F. S. III , & Shaver, G. R. (1985). Individualistic growth responses of tundra plant species to environmental manipulations in the field. Ecology, 66, 564–576. https://doi.org/10.2307/1940405

[ece33939-bib-0012] Chapin, F. S. III , Shaver, G. R. , Giblin, A. E. , Nadelhoffer, K. J. , & Laundre, J. A. (1995). Responses of Arctic tundra to experimental and observed changes in climate. Ecology, 76, 694–711. https://doi.org/10.2307/1939337

[ece33939-bib-0013] Diffenbaugh, N. S. , & Field, C. B. (2013). Changes in ecologically critical terrestrial climate conditions. Science, 341, 486–492. https://doi.org/10.1126/science.1237123 23908225 10.1126/science.1237123

[ece33939-bib-0014] Duursma, R. A. (2015). Plantecophys ‐ An R package for analysing and modelling leaf gas exchange data. PLoS ONE, 10, e0143346 https://doi.org/10.1371/journal.pone.0143346 26581080 10.1371/journal.pone.0143346 PMC4651500

[ece33939-bib-0015] Duursma, R. A. (2016). plantecophys: Modelling and analysis of leaf gas exchange data, R package version 1.1‐8. Retrieved from https://cran.r-project.org/package=plantecophys

[ece33939-bib-0016] EDCT [Environmental Data Center Team] (2017). Meteorological monitoring program at Toolik, Alaska. Toolik Field Station, Institute of Arctic Biology, University of Alaska, Fairbanks, AK. Retrieved from http://toolik.alaska.edu/edc/abiotic_monitoring/data_query.php

[ece33939-bib-0017] Elmendorf, S. C. , Henry, G. H. R. , Hollister, R. D. , Björk, R. G. , Bjorkman, A. D. , Callaghan, T. V. , … Wookey, P. A. (2012). Global assessment of experimental climate warming on tundra vegetation: Heterogeneity over space and time. Ecology Letters, 15, 164–175. https://doi.org/10.1111/j.1461-0248.2011.01716.x 22136670 10.1111/j.1461-0248.2011.01716.x

[ece33939-bib-0018] Etterson, J. R. , & Shaw, R. G. (2001). Constraint to adaptive evolution in response to global warming. Science, 294, 151–154. https://doi.org/10.1126/science.1063656 11588260 10.1126/science.1063656

[ece33939-bib-0019] Evans, J. R. (1989). Photosynthesis and nitrogen relationships in leaves of C_3_ plants. Oecologia, 78, 9–19. https://doi.org/10.1007/BF00377192 28311896 10.1007/BF00377192

[ece33939-bib-0020] Evans, J. R. , & Seemann, J. R. (1989). The allocation of protein nitrogen in the photosynthetic apparatus: Costs, consequences and control In Briggs W. R. (Ed.), Photosynthesis (pp. 183–205). New York, NY: Alan R. Liss.

[ece33939-bib-0021] Farquhar, G. D. , von Caemmerer, S. , & Berry, J. A. (1980). A biochemical model of photosynthetic CO_2_ assimilation in leaves of C_3_ species. Planta, 149, 78–90. https://doi.org/10.1007/BF00386231 24306196 10.1007/BF00386231

[ece33939-bib-0022] Fetcher, N. , & Shaver, G. R. (1982). Growth and tillering patterns within tussocks of *Eriophorum vaginatum* . Holarctic Ecology, 5, 180–186.

[ece33939-bib-0023] Fetcher, N. , & Shaver, G. R. (1983). Life histories of tillers of *Eriophorum vaginatum* in relation to tundra disturbance. Journal of Ecology, 71, 131–147. https://doi.org/10.2307/2259967

[ece33939-bib-0024] Fetcher, N. , & Shaver, G. R. (1990). Environmental sensitivity of ecotypes as a potential influence on primary productivity. The American Naturalist, 136, 126–131. https://doi.org/10.1086/285085

[ece33939-bib-0025] Haugen, R. K. (1982). Climate of remote areas in north‐central Alaska 1975–1979 summary. Hanover, NH: Cold Regions Research and Engineering Lab.

[ece33939-bib-0026] Heskel, M. A. , Bitterman, D. , Atkin, O. K. , Turnbull, M. H. , & Griffin, K. L. (2014). Seasonality of foliar respiration in two dominant plant species from the Arctic tundra: Response to long‐term warming and short‐term temperature variability. Functional Plant Biology, 41, 287–300. https://doi.org/10.1071/FP13137 10.1071/FP13137 32480989

[ece33939-bib-0027] Heskel, M. , Greaves, H. , Kornfeld, A. , Gough, L. , Atkin, O. K. , Turnbull, M. H. , … Griffin, K. L. (2013). Differential physiological responses to environmental change promote woody shrub expansion. Ecology and Evolution, 3, 1149–1162. https://doi.org/10.1002/ece3.525 23762503 10.1002/ece3.525 PMC3678471

[ece33939-bib-0028] Kattge, J. , & Knorr, W. (2007). Temperature acclimation in a biochemical model of photosynthesis: A reanalysis of data from 36 species. Plant, Cell and Environment, 30, 1176–1190. https://doi.org/10.1111/j.1365-3040.2007.01690.x 10.1111/j.1365-3040.2007.01690.x 17661754

[ece33939-bib-0029] Kornfeld, A. , Heskel, M. , Atkin, O. K. , Gough, L. , Griffin, K. L. , Horton, T. W. , & Turnbull, M. H. (2013). Respiratory flexibility and efficiency are affected by simulated global change in Arctic plants. New Phytologist, 197, 1161–1172. https://doi.org/10.1111/nph.12083 23278298 10.1111/nph.12083

[ece33939-bib-0030] Linhart, Y. B. , & Grant, M. C. (1996). Evolutionary significance of local genetic differentiation in plants. Annual Review of Ecology and Systematics, 27, 237–277. https://doi.org/10.1146/annurev.ecolsys.27.1.237

[ece33939-bib-0031] Lloyd, J. , & Taylor, J. A. (1994). On the temperature dependence of soil respiration. Functional Ecology, 8, 315–323. https://doi.org/10.2307/2389824

[ece33939-bib-0032] Mark, A. F. , Fetcher, N. , Shaver, G. R. , & Chapin, F. S. III (1985). Estimated ages of mature tussocks of *Eriophorum vaginatum* along a latitudinal gradient in central Alaska, U.S.A. Arctic and Alpine Research, 17, 1–5. https://doi.org/10.2307/1550957

[ece33939-bib-0033] McGraw, J. B. , Turner, J. B. , Souther, S. , Bennington, C. C. , Vavrek, M. C. , Shaver, G. R. , & Fetcher, N. (2015). Northward displacement of optimal climate conditions for ecotypes of *Eriophorum vaginatum* L. across a latitudinal gradient in Alaska. Global Change Biology, 21, 3827–3835. https://doi.org/10.1111/gcb.12991 26033529 10.1111/gcb.12991

[ece33939-bib-0034] Medlyn, B. E. , Dreyer, E. , Ellsworth, D. , Forstreuter, M. , Harley, P. C. , Kirschbaum, M. U. F. , … Loustau, D. (2002). Temperature response of parameters of a biochemically based model of photosynthesis. II. A review of experimental data. Plant, Cell and Environment, 25, 1167–1179. https://doi.org/10.1046/j.1365-3040.2002.00891.x

[ece33939-bib-0035] NRCS [National Resource Conservation Service] (2017). Daily SNOTEL Data Report ‐ Historic, Coldfoot (SNOTEL 958) and Sagwon (SNOTEL 1183). National Water and Climate Center, U.S. Department of Agriculture. Retrieved from https://www.wcc.nrcs.usda.gov/snow/snotel-data.html

[ece33939-bib-0036] Oberbauer, S. F. , Tweedie, C. E. , Welker, J. M. , Fahnestock, J. T. , Henry, G. H. R. , Webber, P. J. , … Starr, G. (2007). Tundra CO_2_ fluxes in response to experimental warming across latitudinal and moisture gradients. Ecological Monographs, 77, 221–238. https://doi.org/10.1890/06-0649

[ece33939-bib-0037] Onoda, Y. , Hikosaka, K. , & Hirose, T. (2005). The balance between RuBP carboxylation and RuBP regeneration: A mechanism underlying the interspecific variation in acclimation of photosynthesis to seasonal change in temperature. Functional Plant Biology, 32, 903–910. https://doi.org/10.1071/FP05024 10.1071/FP05024 32689186

[ece33939-bib-0038] Parker, T. C. , Tang, J. , Clark, M. B. , Moody, M. M. , & Fetcher, N. (2017). Ecotypic differences in the phenology of the tundra species *Eriophorum vaginatum* reflect sites of origin. Ecology and Evolution, 7, 9775–9786. https://doi.org/10.1002/ece3.3445 29188008 10.1002/ece3.3445 PMC5696421

[ece33939-bib-0039] R Core Team (2016). R: A Language and Environment for Statistical Computing. Vienna, Austria: R Foundation for Statistical Computing.

[ece33939-bib-0040] Rogers, A. , Medlyn, B. E. , Dukes, J. S. , Bonan, G. , von Caemmerer, S. , Dietze, M. C. , … Zaehle, S. (2017). A roadmap for improving the representation of photosynthesis in Earth system models. New Phytologist, 213, 22–42. https://doi.org/10.1111/nph.14283 27891647 10.1111/nph.14283

[ece33939-bib-0041] Rogers, A. , Serbin, S. P. , Ely, K. S. , Sloan, V. L. , & Wullschleger, S. D. (2017). Terrestrial biosphere models underestimate photosynthetic capacity and CO_2_ assimilation in the Arctic. New Phytologist, 216, 1090–1103. https://doi.org/10.1111/nph.14740 28877330 10.1111/nph.14740

[ece33939-bib-0042] Sage, R. F. , & Kubien, D. S. (2007). The temperature response of C_3_ and C_4_ photosynthesis. Plant, Cell and Environment, 30, 1086–1106. https://doi.org/10.1111/j.1365-3040.2007.01682.x 10.1111/j.1365-3040.2007.01682.x 17661749

[ece33939-bib-0043] Shaver, G. R. , Fetcher, N. , & Chapin, F. S. III (1986). Growth and flowering in *Eriophorum vaginatum*: Annual and latitudinal variation. Ecology, 67, 1524–1535. https://doi.org/10.2307/1939083

[ece33939-bib-0044] Souther, S. , Fetcher, N. , Fowler, Z. , Shaver, G. R. , & McGraw, J. B. (2014). Ecotypic differentiation in photosynthesis and growth of *Eriophorum vaginatum* along a latitudinal gradient in the Arctic tundra. Botany‐Botanique, 92, 551–561. https://doi.org/10.1139/cjb-2013-0320

[ece33939-bib-0045] Tape, K. , Sturm, M. , & Racine, C. (2006). The evidence for shrub expansion in Northern Alaska and the Pan‐Arctic. Global Change Biology, 12, 686–702. https://doi.org/10.1111/j.1365-2486.2006.01128.x

[ece33939-bib-0046] van de Weg, M. J. , Fetcher, N. , & Shaver, G. R. (2013). Response of dark respiration to temperature in *Eriophorum vaginatum* from a 30‐year‐old transplant experiment in Alaska. Plant Ecology and Diversity, 6, 377–381.

[ece33939-bib-0047] Walker, M. D. , Wahren, C. H. , Hollister, R. D. , Henry, G. H. R. , Ahlquist, L. E. , Alatalo, J. M. , … Wookey, P. A. (2006). Plant community responses to experimental warming across the tundra biome. Proceedings of the National Academy of Sciences of the United States of America, 103, 1342–1346. https://doi.org/10.1073/pnas.0503198103 16428292 10.1073/pnas.0503198103 PMC1360515

[ece33939-bib-0048] Way, D. A. , & Yamori, W. (2014). Thermal acclimation of photosynthesis: On the importance of adjusting our definitions and accounting for thermal acclimation of respiration. Photosynthesis Research, 119, 89–100. https://doi.org/10.1007/s11120-013-9873-7 23812760 10.1007/s11120-013-9873-7

[ece33939-bib-0049] Yamori, W. , Hokosaka, K. , & Way, D. A. (2014). Temperature response of photosynthesis in C_3_, C_4_, and CAM plants: Temperature acclimation and temperature adaptation. Photosynthesis Research, 119, 101–117. https://doi.org/10.1007/s11120-013-9874-6 23801171 10.1007/s11120-013-9874-6

[ece33939-bib-0050] Yamori, W. , Noguchi, K. , & Terashima, I. (2005). Temperature acclimation of photosynthesis in spinach leaves: Analyses of photosynthetic components and temperature dependencies of photosynthetic partial reactions. Plant, Cell and Environment, 28, 536–547. https://doi.org/10.1111/j.1365-3040.2004.01299.x

